# Supplementary material for: Experience with Rifabutin-Containing Therapy in 500 Patients from the European Registry on Helicobacter pylori Management (Hp-EuReg)
Source: J Clin Med. 2022 Mar 16;11(6):1658. doi: 10.3390/jcm11061658 (PMC8949410; doi:10.3390/jcm11061658)
Supplement: Supplementary file 1 [file jcm-11-01658-s001.zip › jcm-1622333-supplementary.pdf]

Supplementary Table S1. Effectiveness of both triple therapy with amoxicillin and rifabutin and quadruple therapy with amoxicillin, rifabutin and bismuth, according to duration, potency of acid inhibition and treatment line.

				Effectiveness					
**Line***	**Length (days)**	**PPI dose**	**Treatment**	**ITT, N (%)**	**95%CI**	**mITT, N (%)**	**95%CI**	**PP, N (%)**	**95%CI**
**2nd line**	**10**	**low**	**PPI+A+R**	7 (71.4)	29.0-96.3	7 (71.4)	29.0-96.3	7 (71.4)	29.0-96.3
		**standard**	**PPI+A+R**	2 (50.0)	1.2-98.7	2 (50.0)	1.2-98.7	2 (50.0)	1.2-98.7
		**high**	**PPI+A+R**	8 (75.0)	34.9-96.8	7 (85.7)	42.1-99.6	7 (85.7)	42.1-99.6
		**total**	**PPI+A+R**	17 (70.6)	44.0-89.6	16 (75.0)	47.6-92.7	16 (75.0)	47.6-92.7
	**12**	**low**	**PPI+A+R**	59 (57.6)	44.1-71.1	52 (65.4)	51.4-79.2	52 (65.4)	51.4-79.2
		**high**	**PPI+A+R**	72 (76.4)	65.8-86.8	60 (91.7)	81.6-97.2	58 (93.1)	83.3-98.1
		**total**	**PPI+A+R**	131 (67.9)	59.5-76.3	112 (79.5)	71.5-87.3	110 (80.0)	72.1-87.9
	**14**	**low**	**PPI+A+R**	5 (40.0)	5.3-85.3	5 (40.0)	5.3-85.3	5 (40.0)	5.3-85.3
	**Total**	**low**	**PPI+A+R**	71 (57.7)	45.5-69.9	64 (64.1)	51.5-76.6	64 (64.1)	51.5-76.6
		**standard**	**PPI+A+R**	2 (50.0)	1.2-98.7	2 (50.0)	1.2-98.7	2 (50.0)	1.2-98.7
		**high**	**PPI+A+R**	80 (76.3)	66.3-86.2	67 (91)	83.4-98.6	65 (92.3)	82.9-97.4
		**total**	**PPI+A+R**	153 (67.3)	59.5-75.1	133 (77.4)	69.9-84.9	131 (77.9)	70.4-85.3
**3rd line**	**10**	**low**	**PPI+A+R**	4 (100)	39.7-100	4 (100)	39.7-100	4 (100)	39.7-100
		**standard**	**PPI+A+R**	2 (50.0)	1.2-98.7	2 (50.0)	1.2-98.7	2 (50.0)	1.2-98.7
		**high**	**PPI+A+R**	2 (50.0)	1.2-98.7	1 (100)	1.2-98.7	1 (100)	1.2-98.7
		**total**	**PPI+A+R**	8 (75.0)	34.9-96.8	7 (85.7)	42.1-99.6	7 (85.7)	42.1-99.6
	**12**	**low**	**PPI+A+R**	40 (67.5)	51.7-83.2	35 (77.1)	61.8-92.4	35 (77.1)	61.8-92.4
		**high**	**PPI+A+R**	42 (66.7)	51.2-82.1	32 (87.5)	71.0-96.4	31 (87.1)	70.2-96.4
		**total**	**PPI+A+R**	82 (67.1)	56.3-77.8	67 (82.1)	72.1-92.0	66 (81.8)	71.7-91.8
	**14**	**low**	**PPI+A+R**	6 (0.0)	0.0-45.9	4 (0.0)	0.0-60.2	3 (0.0)	0.0-70.8
			**PPI+A+R+B**	1 (100)	1.2-98.7	1 (100)	1.2-98.7	1 (100)	1.2-98.7
		**standard**	**PPI+A+R+B**	2 (100)	15.8-100	2 (100)	15.8-100	2 (100)	15.8-100
		**high**	**PPI+A+R**	11 (36.4)	10.9-69.2	5 (80.0)	28.3-99.5	5 (80.0)	28.3-99.5
		**total**	**PPI+A+R**	17 (23.5)	6.8-49.8	9 (44.4)	13.7-78.8	8 (50.0)	15.7-84.3
			**PPI+A+R+B**	3 (100)	29.2-100	3 (100)	29.2-100	3 (100)	29.2-100
	**Total**	**low**	**PPI+A+R**	50 (62.0)	47.5-76.4	43 (72.1)	57.5-86.6	42 (73.8)	59.3-88.3
			**PPI+A+R+B**	1 (100)	1.2-98.7	1 (100)	1.2-98.7	1 (100)	1.2-98.7
		**standard**	**PPI+A+R**	2 (50.0)	1.2-98.7	2 (50)	1.2-98.7	2 (50.0)	1.2-98.7
			**PPI+A+R+B**	2 (100)	15.8-100	2 (100)	15.8-100	2 (100)	15.8-100
		**high**	**PPI+A+R**	55 (60.0)	46.1-73.8	38 (86.8)	71.9-95.6	37 (86.5)	71.2-95.4
		**total**	**PPI+A+R**	107 (60.7)	51.0-70.5	83 (78.3)	68.8-87.8	81 (79.0)	69.5-88.5
			**PPI+A+R+B**	3 (100)	29.2-100	3 (100)	29.2-100	2 (100)	15.8-100
**4th line**	**10**	**low**	**PPI+A+R**	22 (50.0)	26.8-73.2	21 (52.4)	28.6-76.1	20 (55)	31.5-76.9
			**PPI+A+R+B**	2 (50.0)	1.2-98.7	2 (50.0)	1.2-98.7	2 (50.0)	1.2-98.7
		**standard**	**PPI+A+R**	15 (73.3)	44.9-92.2	15 (73.3)	44.9-92.2	14 (78.6)	49.2-95.3
		**high**	**PPI+A+R**	6 (83.3)	35.9-99.6	5 (100)	47.8-100	5 (100)	47.8-100
			**PPI+A+R+B**	19 (68.4)	43.4-87.4	19 (68.4)	43.4-87.4	18 (66.7)	40.9-86.6
		**total**	**PPI+A+R**	43 (62.8)	47.2-78.4	41 (65.9)	50.1-81.6	39 (69.2)	53.4-84.9
			**PPI+A+R+B**	21 (66.7)	43.0-85.4	21 (66.7)	43.0-85.4	20 (65)	40.7-84.6
	**12**	**low**	**PPI+A+R**	12 (58.3)	27.6-84.8	10 (70.0)	34.7-93.3	10 (70.0)	34.7-93.3
		**high**	**PPI+A+R**	11 (72.7)	39.0-93.9	9 (88.9)	51.7-99.7	9 (88.9)	51.7-99.7
		**total**	**PPI+A+R**	23 (65.2)	43.6-86.8	19 (78.9)	54.4-93.9	19 (78.9)	54.4-93.9
	**14**	**low**	**PPI+A+R**	6 (66.7)	22.2-95.6	6 (66.7)	22.2-95.6	6 (66.7)	22.2-95.6

	**standard**	**PPI+A+R**	3 (33.3)	0.8-90.6	2 (50.0)	1.2-98.7	2 (50.0)	1.2-98.7
	**high**	**PPI+A+R**	21 (14.3)	3.0-36.3	8 (37.5)	8.5-75.5	7 (42.9)	9.8-81.5
	**total**	**PPI+A+R**	30 (26.7)	9.2-44.1	16 (50.0)	24.6-75.3	15 (53.3)	26.6-78.7
**Total**	**low**	**PPI+A+R**	40 (55.0)	38.3-71.7	37 (59.5)	42.2-76.6	36 (61.1)	43.7-78.4
		**PPI+A+R+B**	2 (50.0)	1.2-98.7	2 (50.0)	1.2-98.7	2 (50.0)	1.2-98.7
	**standard**	**PPI+A+R**	18 (66.7)	40.9-86.6	17 (70.6)	44.0-89.6	16 (75.0)	47.6-92.7
	**high**	**PPI+A+R**	38 (42.1)	25.1-59.1	22 (72.7)	49.7-89.2	21 (76.2)	52.8-91.8
		**PPI+A+R+B**	19 (68.4)	43.4-87.4	19 (68.4)	43.4-87.4	18 (66.7)	40.9-86.6
	**total**	**PPI+A+R**	96 (52.1)	41.6-62.6	76 (65.8)	54.4-77.1	73 (68.5)	57.1-79.8
		**PPI+A+R+B**	21 (66.7)	43.0-85.4	21 (66.7)	43.0-85.4	20 (65.0)	40.7-84.6

A: amoxicillin; B: bismuth; R: rifabutin; ITT: intention-to-treat ; PPI: proton pump inhibitor (low-dose PPI: 4.5–27 mg omeprazole equivalents (OE) twice daily (bid) (i.e., 20 mg OE bid), standard-dose PPI: 32–40 mg omeprazole equivalents bid (i.e., 40 mg OE bid), high-dose PPI: 54–128 mg omeprazole equivalents bid (i.e., 60 mg OE bid); mITT: modified intention-to-treat; PP: per protocol, CI: confidence interval, N: total number of patients analysed; *the remaining treatment lines were not reported, as less than 5 patients were included in each subgroup.
